# Super-resolution imaging reveals the nanoscale organization of metabotropic glutamate receptors at presynaptic active zones

**DOI:** 10.1126/sciadv.aay7193

**Published:** 2020-04-15

**Authors:** Sana Siddig, Sarah Aufmkolk, Sören Doose, Marie-Lise Jobin, Christian Werner, Markus Sauer, Davide Calebiro

**Affiliations:** 1 Institute of Pharmacology and Toxicology and Bio-Imaging Center, University of Würzburg, Würzburg, Germany.; 2 Department of Pharmacology, Faculty of Pharmacy, University of Khartoum, Khartoum, Sudan.; 3 Department of Biotechnology and Biophysics, Biocenter, University of Würzburg, Würzburg, Germany.; 4 Department of Neurology & Neurosurgery, Montréal Neurological Institute, McGill University, Montréal, QC H3A 2B4, Canada.; 5 Department of Genetics, Harvard Medical School, Boston, MA 02115, USA.; 6 Institute of Metabolism and Systems Research, University of Birmingham, Birmingham, UK.; 7 Centre of Membrane Proteins and Receptors (COMPARE), Universities of Nottingham and Birmingham, Birmingham, UK.

## Abstract

G protein–coupled receptors (GPCRs) play a fundamental role in the modulation of synaptic transmission. A pivotal example is provided by the metabotropic glutamate receptor type 4 (mGluR4), which inhibits glutamate release at presynaptic active zones (AZs). However, how GPCRs are organized within AZs to regulate neurotransmission remains largely unknown. Here, we applied two-color super-resolution imaging by *direct* stochastic optical reconstruction microscopy (*d*STORM) to investigate the nanoscale organization of mGluR4 at parallel fiber AZs in the mouse cerebellum. We find an inhomogeneous distribution, with multiple nanodomains inside AZs, each containing, on average, one to two mGluR4 subunits. Within these nanodomains, mGluR4s are often localized in close proximity to voltage-dependent Ca_V_2.1 channels and Munc-18-1, which are both essential for neurotransmitter release. These findings provide previously unknown insights into the molecular organization of GPCRs at AZs, suggesting a likely implication of a close association between mGluR4 and the secretory machinery in modulating synaptic transmission.

## Teaser

Super-resolution imaging reveals close association between mGluR4, Ca_V_2.1, and Munc-18-1 at cerebellar active zones.

## INTRODUCTION

Chemical synaptic transmission occurs at highly specialized presynaptic structures, known as active zones (AZs), which are the sites where neurotransmitters are stored and released (*1*). The structure and function of AZs vary considerably among different types of neurons or even between synapses of the same neuron (*2*, *3*). Moreover, dynamic changes in the composition, structure, and activity of AZs are believed to be responsible for synaptic plasticity (*4*).

Presynaptic AZs accomplish four main functions in neurotransmitter release (*1*). First, they are responsible for efficient docking, priming, and exocytosis of synaptic vesicles, where neurotransmitters are stored. Second, they guarantee fast and efficient coupling between excitation, Ca^2+^ influx, and neurotransmitter release, which is believed to occur via a tight spatial arrangement of voltage-dependent calcium channels (VDCCs) so that they are located in close proximity to the secretory machinery. Third, they interact via trans-synaptic cell adhesion molecules with the postsynaptic membrane to ensure a precise juxtaposition of AZs and the specialized postsynaptic structures containing neurotransmitter receptors. Last, they represent the main site of regulation of neurotransmitter release, being largely responsible for both short- and long-term synaptic plasticity.

G protein–coupled receptors (GPCRs)—the largest family of receptors for hormones and neurotransmitters—are highly expressed in the central nervous system (CNS), where they play important roles in both short- and long-term modulation of synaptic transmission (*5*). Although GPCRs are expressed both pre- and post-synaptically and can both potentiate and depress synaptic transmission, presynaptic receptors coupled to G_i/o_ proteins exert a critical inhibitory function by acting as either autoreceptors for the locally released neurotransmitters or heteroreceptors for neurotransmitters and neuromodulators released by other neurons (*5*). Despite a wealth of electrophysiological data demonstrating rapid effects of GPCRs on neurotransmitter release (*6*, *7*), the underlying molecular mechanisms are highly debated (*8*–*10*). Whereas some studies suggest that GPCR effects might be mediated via classical second messenger–dependent pathways, others point to a major involvement of Gβγ subunits released upon GPCR activation, which can inhibit VDCCs (Ca_V_2.1 and Ca_V_2.2) and activate G protein–coupled inwardly rectifying potassium (GIRK) channels (*8*, *11*, *12*). Furthermore, there is evidence that GPCRs might regulate VDCCs and/or the secretory machinery through G protein–independent mechanisms, which might involve direct protein-protein interactions. However, only limited information is available on the spatial organization of GPCRs within AZs (*13*–*15*).

The metabotropic glutamate receptor type 4 (mGluR4) is a prototypical presynaptic GPCR that functions as an inhibitory autoreceptor for glutamate, the main excitatory neurotransmitter in the CNS (*16*). mGluR4 is present at particularly high levels in the cerebellum, where it is expressed in granule cells, the axons of which—known as parallel fibers—form dense and highly organized synapses with cerebellar Purkinje cells (*17*–*19*). These synapses provide the link between the major input pathway and the exclusive output pathway of the cerebellar network (*20*). mGluR4 signaling at parallel fiber synapses is important for neuronal survival and normal motor performance (*21*, *22*). Consistently, mGluR4-deficient mice exhibit abnormalities in short-term synaptic plasticity (*23*). Previous data based on immunofluorescence and electron microscopy (EM) indicate that mGluR4 is highly enriched at parallel fiber AZs (*17*, *19*, *24*). Moreover, in vitro data suggest that members of the mGluR family function as a dimer containing two receptor subunits and might form higher-order oligomers that could regulate its function (*25*–*27*). However, the spatial arrangement and stoichiometry of mGluR4 complexes within cerebellar AZs in vivo is presently largely unknown.

Despite an ever-growing knowledge of the individual proteins involved in the structure and function of AZs, little is known about their spatial organization within intact AZs, which have a size of about 200 to 400 nm at a central synapse (*28*). This is mostly due to technical limitations of conventional microscopy methods, which have a spatial resolution limit of about 200 nm. However, recently developed super-resolution microscopy techniques (*29*, *30*), which can achieve a lateral resolution of 10 to 20 nm, provide a unique opportunity to directly image the nanoscale organization of AZs in native cells and tissues and, thus, investigate some of the most fundamental mechanisms at the basis of neurotransmitter release and its regulation.

In this study, we use a super-resolution microscopy method based on single-molecule localization [*direct* stochastic optical reconstruction microscopy (*d*STORM)] (*31*, *32*), combined with single-molecule measurements under controlled conditions (*33*, *34*), to obtain a detailed nanoscopic characterization of the spatial arrangement and stoichiometry of mGluR4s at parallel fiber AZs in the mouse cerebellum. We find a high degree of organization, with each AZ containing, on average, approximately 35 mGluR4 subunits, arranged in small nanodomains and often in close proximity to Munc-18-1 and Ca_V_2.1 channels. Our data provide previously unknown insights into the ultrastructural organization of mGluR4 receptors at parallel fiber AZs, which improve our understanding of the mechanisms underlying the rapid regulation of neurotransmitter release by GPCRs.

## RESULTS

### *d*STORM imaging of mGluR4 at cerebellar presynaptic AZs

**Fig. 1:**
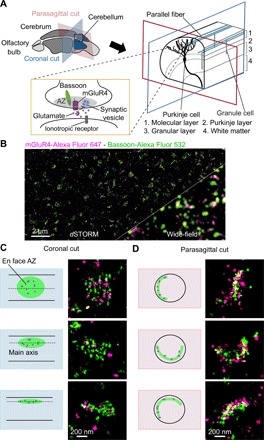
Super-resolution (*d*STORM) imaging reveals the nanoscale organization of mGluR4 at parallel fiber AZs. (**A**) Schematic view of the organization of the mouse cerebellum, showing the ordered arrangement of the parallel fibers, which originate from granule cells and form dense synapses with the dendritic spines of Purkinje cells. mGluR4s located on the presynaptic membrane of parallel fiber synapses regulate synaptic transmission by inhibiting the release of glutamate (yellow box). Two different planes were used for cutting the cerebellum: coronal (blue; parallel to parallel fibers) and parasagittal (red; perpendicular to parallel fibers). (**B**) Two-color *d*STORM imaging of mGluR4 (magenta) and bassoon (green). An image of a coronal section acquired in a region corresponding to the molecular layer of the cerebellum is shown. The corresponding wide-field fluorescence image is given for comparison. (**C** and **D**) Enlarged views of representative AZs imaged by *d*STORM in either coronal (C) or parasagittal (D) sections. Note the different orientations of the AZs relative to the imaging plane. AZs captured en face as in the top example in (C) were used in subsequent analyses. Images in (C) and (D) are representative of two and four independent experiments, respectively.

To analyze the subcellular distribution of mGluR4 at presynaptic AZs, we imaged the outer layer of the cerebellar cortex (molecular layer), where axons of granule cells form dense synapses with the dendritic spines of Purkinje cells (Fig. 1A). To obtain images of the AZs found at these synapses with a defined orientation, we took advantage of the unique parallel arrangement of granule cell axons—known as parallel fibers—which we cut along two defined planes: coronal and parasagittal, i.e., parallel or perpendicular to parallel fibers, respectively (Fig. 1A). mGluR4 was labeled with a specific polyclonal antibody raised against a 23–amino acid–long C-terminal epitope (*17*, *35*). The specificity of the mGluR4 antibody was verified in mGluR4 knockout mice (*35*). AZs were identified by simultaneously labeling bassoon, a scaffold protein that plays an important role in the organization of AZs and the recruitment of presynaptic vesicles (*1*). Wide-field images showed colocalization between mGluR4 and bassoon but could not resolve their distribution within AZs (Fig. 1B), as these have a diameter of approximately 200 to 400 nm (*28*), which roughly corresponds to the resolution limit of fluorescence microscopy. Therefore, we took advantage of the super-resolution capabilities of *d*STORM, which allowed us to image and analyze the distribution of mGluR4 at AZs with a localization precision of approximately 13 nm on average (fig. S1A). The *d*STORM images revealed a high density of mGluR4 localizations within AZs (Fig. 1B). As expected, AZ images acquired from coronal sections typically had elliptical shapes with different degrees of elongation, corresponding to AZs captured with their main axis parallel to the imaging plane and different positions around parallel fibers (Fig. 1C). Instead, AZ images acquired from parasagittal sections typically had a sickle-like appearance, corresponding to side views of AZs having their main axis perpendicular to the imaging plane (Fig. 1D). Subsequent analyses were performed on images of en face AZ obtained from coronal sections, which were automatically identified based on their shape (see Materials and Methods and fig. S2A for details).

### Spatial organization and clustering of mGluR4 at cerebellar AZs

**Fig. 2:**
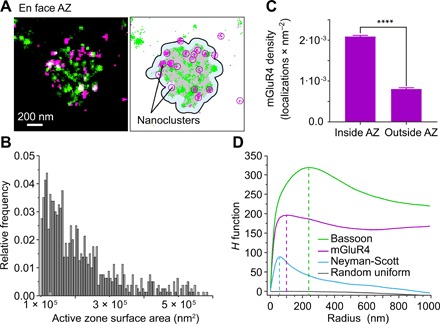
Organization of mGluR4 at parallel fiber AZs. (**A**) Principle of the analysis. En face AZs were identified on the basis of the bassoon localizations (green dots) using the DBSCAN algorithm (*53*). Gray color indicates the AZ area identified by the analysis. mGluR4 nanoclusters (magenta circles) were subsequently identified on the basis of the mGluR4 localizations (magenta dots) using DBSCAN (see fig. S1 for details). (**B**) Histogram reporting the surface areas of the analyzed en face AZs. (**C**) Comparison of mGluR4 localization densities inside and outside AZs. *n* = 799 AZs. Data are means ± SEM. ****P < 0.0001 by two-sided paired *t* test. (**D**) Ripley’s *H* function analysis investigating the clustering of mGluR4 and bassoon on different length scales. Data were compared with a Neyman-Scott distribution (*n* = 20 and σ = 20 nm) to simulate randomly distributed localization clusters and with random uniformly distributed localizations. *H* maxima were observed at approximately 240 nm (bassoon), 100 nm (mGluR4), and 60 nm (Neyman-Scott).

We then investigated the spatial organization and clustering of mGluR4 at parallel fiber AZs, which we identified automatically based on bassoon localizations using the clustering algorithm DBSCAN (Fig. 2A; see Materials and Methods and fig. S1 for details) (*36*). The surface area of AZs exhibited a broad distribution (mean, 2.16 ± 0.03 × 10^5^ nm^2^; range, 1.00 × 10^5^ to 5.81 × 10^5^ nm^2^) (Fig. 2B), consistent with previous measurements based on freeze-fracture replica EM (*37*). Most mGluR4 localizations were found within AZs, as defined by the presence of bassoon. On the basis of their localization densities, we calculated that mGluR4s were about four times more concentrated inside AZs compared to outside (2.1 ± 0.4 × 10^−3^ versus 5.8 ± 0.4 × 10^−4^ localizations/nm^2^) (Fig. 2C), in agreement with previous results (*38*). To evaluate whether the distribution of mGluR4 differed between the center and periphery of AZs, we computed the distance of each mGluR4 localization from the AZ border. For some AZs, we observed a preferential localization of mGluR4s at their periphery. However, the average distribution of mGluR4 from the AZ border toward its center did not differ substantially from what is expected for a similar number of random localizations, suggesting the lack of a general gradient between center and periphery and/or a high variability in the distribution among AZs (fig. S2, B to D).

Within AZs, mGluR4 localizations were apparently concentrated in small nanoclusters with a size in the range of the spatial resolution provided by *d*STORM (Fig. 2A). Multiple phenomena can contribute to the occurrence of these nanoclusters. First, more than one secondary antibody can bind to each primary antibody. Second, each secondary antibody often carries more than one fluorophore and the fluorophores used in *d*STORM (e.g., Alexa Fluor 647) typically blink several times before bleaching, producing multiple localizations for the same fluorophore. Third, and most importantly, more than one receptor might be present at a distance below the resolution limit, as would be expected in the case of supramolecular complexes. Therefore, any quantitative interpretation of *d*STORM data requires a precise analysis of the nature of these nanoclusters.

To obtain an initial characterization of mGluR4 clustering on different length scales, we computed the Ripley’s *H* function (*39*) of mGluR4 localizations, comparing it with that obtained for bassoon or simulated localizations (Fig. 2D). The Ripley’s *H* function obtained for mGluR4 displays a bimodal shape, with a main peak around 100 nm and a shoulder around 240 nm (Fig. 2D), indicating higher-order clustering. Because a broad peak around 240 nm can also be observed in the distribution of the bassoon localizations (Fig. 2D), the shoulder in the Ripley’s *H* function of mGluR4 localizations could be explained by mGluR4 preferential location within AZs. To better interpret the peak around 100 nm, we considered the case of randomly distributed localization clusters, which we simulated as a Neyman-Scott process with 20 localizations per cluster and an SD (σ) of 20 nm (the typical spatial resolution of *d*STORM experiments, considering the localization precision and size of antibodies). This simulated distribution gave a peak around 60 nm, which is substantially lower than the value of 100 nm measured for mGluR4. Thus, the occurrence of multiple localizations for a single mGluR4 subunit appeared insufficient to fully explain the characteristics of the first peak in the mGluR4 distribution. Together, these data were consistent with mGluR4 having a nonrandom distribution within AZs.

### Number and size of mGluR4 nanoclusters within AZs

**Fig. 3:**
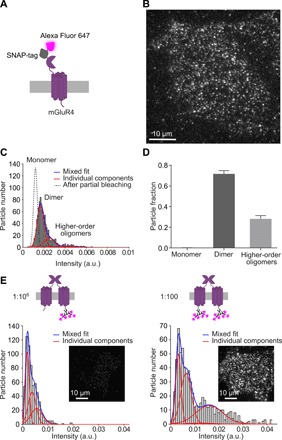
Analysis of mGluR4 stoichiometry by single-molecule microscopy. (**A**) Schematic view of the mGluR4 construct carrying an N-terminal SNAP-tag (SNAP-mGluR4), which was used for the analysis. The construct was transiently expressed in CHO cells at low densities, corresponding to 0.45 ± 0.08 (SD) fluorescently labeled mGluR4s/μm^2^, and labeled at 1:1 stoichiometry with a saturating concentration of an Alexa Fluor 647 benzylguanine derivative, which binds covalently and irreversibly to the SNAP-tag. Cells were sequentially fixed and imaged by TIRF microscopy. (**B**) Representative TIRF image of a fixed CHO cell expressing the SNAP-mGluR4 construct. Dots represent individual receptor particles, which were identified with an automated single-particle detection algorithm. (**C**) Representative distribution of the intensity of mGluR4 particles in a cell expressing the SNAP-mGluR4 construct. Data were fitted with a mixed Gaussian model. The result of a mixed Gaussian fitting after partial photobleaching (dotted black line) was used to precisely estimate the intensity of single fluorophores in each image sequence. a.u., arbitrary units. (**D**) Relative abundance of monomers, dimers, and higher-order oligomers or nanoclusters detected by the analysis. Data are means ± SEM of 11 cells from three independent experiments (12,012 particles). (**E**) Estimation of the number of primary antibodies binding to one mGluR4. CHO cells transiently transfected to express wild-type mGluR4 at low densities—0.55 ± 0.07 (SD) fluorescently labeled mGluR4s/μm^2^—were incubated with either a limiting dilution (1:10^6^) or a saturating concentration (1:100) of the primary antibody against mGluR4 and labeled with an Alexa Fluor 647–conjugated secondary antibody. Cells were then imaged and analyzed as in (B) and (C). Representative images and results of 20 (17,257) and 22 (13,553) cells from three independent experiments, respectively (number of particles in brackets), are shown.

We then sought to estimate the number of mGluR4 present within an AZ and within a localization nanocluster. Previous studies indicated that mGluR subunits form homo- and heterodimers and possibly higher-order oligomers, at least partially mediated by intersubunit disulfide bridges, and that dimerization is required for their function (*40*, *41*). mGluR2 receptors have also been shown to form homodimers in cultured hippocampal neurons and to form larger oligomers upon both receptor activation and inhibition (*27*). However, the supramolecular organization of mGluR4 in native tissue is largely unknown. To gain further insights into the supramolecular organization of mGluR4, we first applied single-molecule microscopy to mGluR4s expressed in a simple cell system under controlled conditions (*33*, *34*). For this purpose, we expressed SNAP-tagged mGluR4s in Chinese hamster ovary (CHO) cells at low/physiological densities, i.e., 0.45 ± 0.08 (SD) fluorescently labeled mGluR4s/μm^2^, and covalently labeled them with a bright organic fluorophore (Alexa Fluor 647) via the SNAP-tag (Fig. 3A). Individual mGluR4 complexes were then imaged by total internal reflection fluorescence (TIRF) microscopy in fixed cells (Fig. 3B). A mixed Gaussian fitting algorithm on the distribution of particle intensities was used to estimate the relative abundance of monomers, dimers, and higher-order oligomers/nanoclusters, as previously described (*33*). The results indicated that most mGluR4 particles contained two subunits (70%), with a minor fraction containing three or more subunits (Fig. 3, C and D). The ability of our single-molecule analysis method to precisely analyze the size of the receptor complexes was verified using constructs of the monomeric membrane receptor CD86 with either one or two SNAP-tags fused to its N terminus (fig. S3), which served as controls for monomers and dimers, respectively (*33*).

Knowing the supramolecular organization of mGluR4s under these controlled conditions also allowed us to estimate the number of primary antibodies binding to one mGluR4 under the same experimental conditions. To this end, we labeled CHO cells expressing mGluR4 at similarly low/physiological densities, i.e., 0.55 ± 0.07 (SD) fluorescently labeled mGluR4s/μm^2^, with either limiting dilution or saturating concentrations of the primary antibody. We then compared the distributions of the intensities of the corresponding particles obtained by TIRF microscopy (Fig. 3E). The differences observed between the two distributions were indicative of the binding of one primary antibody per mGluR4 (see Supplementary Results for details). Binding of one primary antibody per mGluR4 was also consistent with the short length (23 amino acids) of the epitope used to raise the primary antibody (*17*, *35*).

Having obtained these data, we set out to estimate the number of mGluR4 subunits present in parallel fiber AZs and within AZ nanoclusters, which we identified in *d*STORM images by DBSCAN as shown in fig. S1. For this purpose, we performed *d*STORM imaging at various concentrations of the primary antibody while measuring the mean number of localizations per nanocluster (fig. S4) (*42*). We observed a sigmoidal concentration dependence, with two plateaus corresponding to a minimum and maximum number of primary antibodies bound (fig. S4A, right). Nonspecific adsorption of the secondary antibody was negligible, contributing to no more than approximately 0.2% of the localizations detected at 1:100 dilution or 3% of those detected at 1:20,000 dilution, as determined in samples in which the primary antibody was omitted (fig. S4, A and B). Fitting the data with a logistic function, we estimated 20.8 ± 1.0 localizations per nanocluster under saturating conditions $\left(N_{\text{NC},\text{max}}^{\text{mGluR}4}\right)$ and 14.9 ± 0.8 localizations per nanocluster at limiting dilution $\left(N_{\text{NC},\text{min}}^{\text{mGluR}4}\right)$. By dividing these two values, we estimated that each nanocluster contained, on average, 1.4 mGluR4 subunits. Because this estimate is based on the relative numbers of localizations at saturating conditions compared to limiting dilution of the primary antibody, both of which are obtained in the presence of the same secondary antibody, this approach is less sensitive to variability in the number of secondary antibodies per primary antibody or in the number of fluorophores per secondary antibody than other approaches based on absolute numbers of localizations. Moreover, the distribution obtained at saturating conditions was broader than the one obtained at limiting dilution (fig. S4C), consistent with the presence of a variable number of receptor subunits in each nanocluster. Analysis of the data obtained in the absence of primary antibody gave a mean value of 9.8 ± 1.3 localizations per nanocluster (fig. S1B), indicative of an average binding of one to two secondary antibodies per primary antibody and receptor.

To estimate the number of mGluR4 subunits within each AZ, we also calculated the number of mGluR4 localizations per en face AZ $\left(N_{\text{AZ}}^{\text{mGluR}4}\right)$ identified via bassoon staining. This number varied considerably among individual AZs (fig. S5) with a mean value of 522 ± 13 localizations per AZ. On the basis of these data, we estimated that one parallel fiber AZ contains, on average, 25 mGluR4 nanodomains (obtained by dividing $N_{\text{AZ}}^{\text{mGluR}4}$ by $N_{\text{NC},\text{max}}^{\text{mGluR}4}$), each comprising mainly one or two mGluR4 subunits, with few nanodomains possibly comprising three or more receptor subunits. This corresponds, on average, to the presence of approximately 35 mGluR4 subunits per AZ (estimated by dividing $N_{\text{AZ}}^{\text{mGluR}4}$ by $N_{\text{NC},\text{min}}^{\text{mGluR}4}$).

### Arrangement of mGluR4 relative to bassoon and Ca_V_2.1

We then investigated the spatial distribution of mGluR4 relative to that of bassoon and Ca_V_2.1, which is the predominant VDCC in the cerebellum (*43*).

**Fig. 4:**
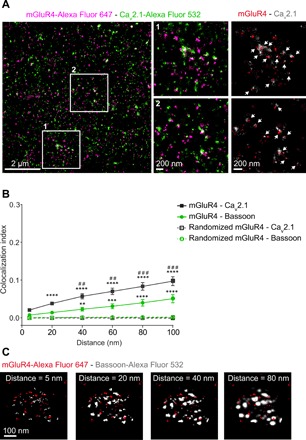
Arrangement of mGluR4 relative to bassoon and Ca_V_2.1 channels by distance-based colocalization analysis. (**A**) Two-color *d*STORM imaging of mGluR4 (magenta) and Ca_V_2.1 channels (green). Left: Representative super-resolved *d*STORM image revealing the organization of mGluR4 relative to Ca_V_2.1. Middle: Enlarged views corresponding to the regions delimited by the white boxes. Right: Images of same regions, where mGluR4 localizations are shown in red over Ca_V_2.1 localizations in gray (areas with no or low mGluR4 localization densities, corresponding to sites outside AZs, are not shown). White arrows, areas of colocalization between mGluR4 and Ca_V_2.1. (**B**) Distance-based colocalization analysis. The colocalization index values calculated over increasing distance, corresponding to the SD of the Gaussian filter applied to the bassoon or Ca_V_2.1 channel, are reported. Results were compared to those obtained with an equal number of random uniformly distributed mGluR4 localizations (dashed lines). Data are means ± SEM of 7 or 10 *d*STORM images from two independent preparations each, coimmunostained for mGluR4 and bassoon or mGluR4 and Ca_V_2.1, respectively. Differences are statistically significant by two-way ANOVA followed by Holm-Sidak’s test. (**C**) Principle of the analysis. A Gaussian filter with increasing SD is applied to the bassoon channel, allowing the estimation of colocalization between mGluR4 and bassoon over increasing distances (see Materials and Methods for details). **P < 0.01, ***P < 0.001, and ****P < 0.0001 versus random localizations. ^##^*P* < 0.01 and ^###^*P* < 0.001 versus mGluR4-bassoon.

First, we evaluated the distribution of Ca_V_2.1 relative to bassoon by two-color *d*STORM. The results revealed a partial enrichment of Ca_V_2.1 at parallel fiber AZs (fig. S6). However, Ca_V_2.1 channels were also found outside AZs (fig. S6), consistent with the occurrence of Ca_V_2.1 at extrasynaptic sites on parallel fibers and on dendrites of Purkinje cells (*37*). Next, we costained cerebellar slices for mGluR4 and Ca_V_2.1 and investigated them by *d*STORM, as done for mGluR4 and bassoon. The obtained *d*STORM images indicated that Ca_V_2.1 channels were often present at cerebellar AZs in close proximity to mGluR4s (Fig. 4A).

We then took advantage of the localizations obtained by two-color *d*STORM to perform a distance-based colocalization analysis, providing quantitative information about the arrangement of mGluR4 relative to either bassoon or Ca_V_2.1 on different spatial scales (Fig. 4, B and C; see Materials and Methods for details).

To validate the method, we first stained cerebellar slices using two different primary antibodies against bassoon, which were raised in different species, where maximal colocalization was expected. The analysis gave colocalization indexes between approximately 0.2 (evaluated at 20 nm) and 0.6 (at 100 nm) (fig. S7), which served as a reference for subsequent analyses. This increase of the colocalization index with distance scale is a consequence of both the localization error and the physical size of the used antibodies, typically about 10 nm (*44*). Consequently, even in the case of two antibodies binding to the same protein and recognized by different secondary antibodies, the resulting localizations can fall as far apart as about 20 to 30 nm. As a control, replacing one of the channels with a random uniform distribution, a Neyman-Scott distribution, or flipping the second channel all produced negligible colocalization index values (fig. S7, B to D).

We then applied the distance-based colocalization analysis to mGluR4 and either bassoon or Ca_V_2.1. The results revealed a positive correlation between mGluR4 and bassoon localizations on a 40- to 100-nm scale, as indicated by positive colocalization index values (Fig. 4B). This positive correlation was not due to chance, as colocalization values were statistically different from those obtained using an equivalent number of random localizations, either uniformly distributed (Fig. 4B) or following a Neyman-Scott distribution (fig. S8). Despite Ca_V_2.1 being less enriched at AZs than bassoon, an even higher positive correlation was observed when analyzing mGluR4 relative to Ca_V_2.1 instead of bassoon (Fig. 4B and fig. S8). In the case of mGluR4 and Ca_V_2.1, colocalization index values were statistically different from both random distributions already at 20 nm—i.e., at a distance below the localization uncertainty introduced by the antibodies and the localization error—indicating that at least a fraction of mGluR4 and Ca_V_2.1 molecules are located at such short distances to enable physical interactions.

### Nearest neighbor analysis of mGluR4 and Ca_V_2.1

To further investigate the relative distance between mGluR4 and Ca_V_2.1, we used the localization data obtained by two-color *d*STORM to perform a centroid nearest neighbor (NN) analysis (fig. S9). For this purpose, both mGluR4 and Ca_V_2.1 nanoclusters were identified using DBSCAN, and the NN values of the corresponding centroids between the two populations were calculated. As a control, we used randomized positions of the Ca_V_2.1 localization centroids. The analysis gave peaks at approximately 65 and 120 nm for mGluR4 with Ca_V_2.1 and control, respectively (fig. S9D). The results of this analysis further confirmed that mGluR4 and Ca_V_2.1 were closer than it would be expected for a random distribution and that a relevant fraction of mGluR4s and Ca_V_2.1 channels were at a distance that was indicative of close proximity, given the uncertainty due to antibody staining and the two-channel localization error of approximately 20 nm.

### Arrangement of mGluR4 relative to Munc-18-1

Previous studies reported that mGluR4 might directly interact with Munc-18-1 (*15*), a key accessory component of the synaptic secretory machinery that regulates synaptic transmission and is essential for neurotransmitter release. Therefore, we additionally investigated the proximity between mGluR4 and Munc-18-1 using the same approach as for bassoon and Ca_V_2.1 channels.

**Fig. 5:**
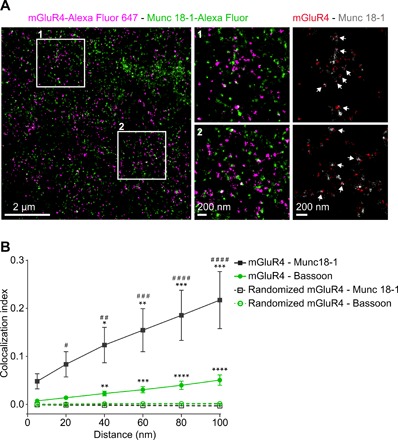
Arrangement of mGluR4 relative to bassoon and Munc-18-1 by distance-based colocalization analysis. (**A**) Representative two-color *d*STORM imaging of mGluR4 (magenta) and Munc-18-1 (green). Left: Super-resolved *d*STORM image revealing the organization of mGluR4 relative to Munc-18-1. Middle: Enlarged views corresponding to the regions delimited by the white boxes. Right: Images of same regions, where mGluR4 localizations are shown in red over Munc-18-1 localizations in gray (areas with no or low mGluR4 localization densities, corresponding to sites outside AZs, are not shown). White arrows, areas of colocalization between mGluR4 and Munc-18-1. (**B**) Distance-based colocalization analysis. Data shown are means ± SEM of seven or three *d*STORM images from two independent preparations coimmunostained for mGluR4 and bassoon and one preparation coimmunostained for mGluR4 and Munc-18-1, respectively. Results were compared to those obtained with an equal number of random uniformly distributed mGluR4 localizations (dashed lines). Differences are statistically significant by two-way ANOVA followed by Holm-Sidak’s test. *P < 0.05, **P < 0.01, ***P < 0.001, and ****P < 0.0001 versus random localizations. ^#^*P* < 0.05, ^##^*P* < 0.01, ^###^*P* < 0.001, and ^####^*P* < 0.0001 versus mGluR4-bassoon.

Two-color *d*STORM imaging revealed that at least a fraction of mGluR4 and Munc-18-1 were present in close proximity at parallel fiber AZs (Fig. 5A). The distance-based colocalization analysis revealed an even stronger association of mGluR4 with Munc-18-1 than with either bassoon or Ca_V_2.1 channels (Fig. 5B and fig. S8). A close proximity between mGluR4 and Munc-18-1 was further supported by a NN analysis, which showed, after compensation for the random component, a distinct distance peak at around 30 nm, which corresponds to the uncertainty of our method (fig. S10). Together, these results point to a close association between at least a fraction of mGluR4 and Munc-18-1, within distances allowing a direct or indirect interaction between the two molecules.

## DISCUSSION

**Fig. 6:**
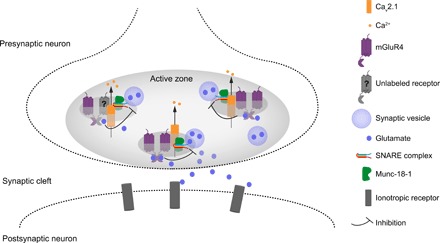
Schematic representation of mGluR4 nanoscale organization within the AZ. Our data reveal a high level of spatial organization at parallel fiber AZs, where we find mGluR4 in close proximity to Ca_V_2.1 channels and Munc-18-1. This places mGluR4 right next to both the channels implicated in calcium influx (Ca_V_2.1) and a key regulator of the SNARE complex (Munc-18-1), which, upon calcium entry, is responsible for the fusion of synaptic vesicles and the resulting release of neurotransmitters. These findings provide an ultrastructural basis to understand the mechanisms implicated in the regulation of synaptic transmission by mGluR4 and possibly other presynaptic GPCRs.

Our study provides a detailed characterization of the number, spatial organization, and stoichiometry of mGluR4—a prototypical presynaptic GPCR—at a model AZ within the CNS. Our results indicate that mGluR4 is highly enriched at parallel fiber AZs, which we show to contain, on average, approximately 35 mGluR4 subunits each. We find mGluR4 to be organized in small nanodomains mainly containing one to two receptor subunits, with few possibly containing three or more subunits. Our data indicate that, within these nanodomains, at least a fraction of mGluR4s are distinctively located in close proximity to Munc-18-1 and Ca_V_2.1 channels. This suggests a possible mechanism for the rapid regulation of neurotransmitter release by mGluR4s, whereby their close association with Ca_V_2.1 channels and the secretory machinery might be able to directly influence Ca^2+^ influx and/or vesicle docking and fusion (Fig. 6). With the exception of few data based on pioneering EM experiments (*17*–*19*, *24*), little information is available about the number, supramolecular state, and spatial arrangement of GPCRs within AZs. Thus, our data provide previously unknown, important insights into the nanoscopic organization of GPCRs at central synapses under physiological conditions.

Conventional light microscopy provides insufficient spatial resolution to investigate the structure of AZs, which have a typical size of 200 to 400 nm (*28*). Therefore, EM has been instrumental to reveal the complex and ordered arrangement of filamentous structures and synaptic vesicles within AZs (*28*, *45*, *46*). Moreover, immunogold labeling enables to locate specific proteins in electron micrographs with nanometer precision. However, specific labeling with antibody-conjugated gold particles is generally inefficient, and there is a trade-off between the achievable tissue preservation and spatial resolution. Thus, there is an urgent need to develop methods that combine the efficient labeling and good structural preservation of immunofluorescence microscopy with the high spatial resolution of EM. Our study demonstrates how *d*STORM can be successfully used as a powerful method to investigate the ultrastructural organization of GPCRs within AZs.

Although the possible clustering of mGluR4 had been suggested in a previous EM study (*24*), our data obtained by *d*STORM under efficient labeling and controlled conditions provide strong evidence for the existence of mGluR4 nanodomains in vivo and allowed us to estimate their number, size, and location. Our data indicate that the distance between mGluR4s within the AZ nanodomains identified by our study is below the spatial resolution of *d*STORM of approximately 20 nm in the lateral direction, which is what is expected in the case of receptor dimers. The formation of mGluR4 dimers is further supported by our single-molecule data obtained in a simple cell system under controlled conditions as well as by in vitro data (*25*) and the results of recent experiments in cultured hippocampal neurons (*27*). Together, these studies strongly suggest that mGluRs assemble into functional dimers, mainly via the formation of intersubunit disulfide bridges, and possibly higher-order oligomers or nanoclusters (*25*, *26*, *47*). Thus, our study provides important direct support for the existence of endogenous mGluR4 dimers in the CNS.

Our experiments also reveal a large variability in the number of mGluR4s contained in parallel fiber AZs and within the nanodomains identified inside them. However, because immunolabeling is restricted to antibody-accessible epitopes, our estimates might represent lower bounds of the actual numbers of mGluR4s. In particular, we cannot rule out that the presence of another interacting protein might interfere with the binding of the used primary antibody. The fact that a fraction of the identified nanodomains appears to contain only one mGluR4 subunit might additionally be explained by either the presence of a subpopulation of monomers, although this subpopulation was not detected in CHO cells visualized by single-molecule microscopy, or by heterodimerization with other members of the mGluR family (*48*), which are not recognized by the antibody used in our study. Because heterodimerization has been suggested to modify the activity of GPCRs (*40*), this might provide an additional mechanism to modulate the function of mGluR4s at AZs. Intriguingly, a recent report in hippocampal neurons has shown that mGluR2 stimulation can promote receptor oligomerization (*27*). Thus, it is possible that the variability in the number of mGluR4s per nanodomain that we observe in cerebellar AZs might also be related to their activity. A similarly high variability has been previously found for Ca_V_2.1 channels (*37*), suggesting the possible existence of AZs in different functional states. Additional studies will be required to investigate the dependency on activity and possible functional relevance of the observed variability in the number of mGluR4s at parallel fiber AZs.

A major finding of our study is the proximity of mGluR4 to both Ca_V_2.1 channels and Munc-18-1 at distances that are expected for physically interacting proteins labeled with pairs of primary and secondary immunoglobulin G antibodies, each with a size of ~10 nm. Our data are consistent with the presence of mGluR4s, Ca_V_2.1 channels, and Munc-18-1 proteins in either small nanodomains or macromolecular complexes. Whereas no direct association between mGluR4 and VDCC has been reported so far, previous studies suggested that postsynaptic mGluR1s might be able to directly interact with Ca_V_2.1 at dendrites of Purkinje neurons to temporarily modulate their activity (*49*). Similarly, the β_2_-adrenergic receptor has been suggested to form a macromolecular signaling complex with Ca_V_1.2, Gα_s_ protein, and adenylyl cyclase (*50*). Intriguingly, a study on mGluR7 also indicates that interaction with the PDZ domain containing protein PICK1, which has been suggested to induce mGluR7 anchoring and clustering (*51*) within AZs, is required for mGluR7-mediated inhibition of VDCCs (*52*). Thus, it is tempting to speculate that the observed close proximity between mGluR4 and Ca_V_2.1 found in our study might play an important role in assuring efficient and specific functional coupling of mGluR4 to Ca_V_2.1 and, hence, in mediating the inhibitory role of mGluR4 on neurotransmitter release (Fig. 6) (*53*, *54*). Moreover, the close proximity revealed by our study between mGluR4 and Munc-18-1 suggests the possibility that mGluR4 might interact with Munc-18-1 to modulate its function, which is essential for vesicle docking and release (Fig. 6). Most recent models suggest that Munc-18-1 participates in the regulation of SNARE complex formation by interacting with the t-SNARE syntaxin 1 in its close/inactive conformation and subsequently favoring the formation of the SNARE complex and stabilizing it (*55*, *56*). Thus, it is tempting to speculate that mGluR4, by interacting with Munc-18-1, might be able to modulate the formation of the SNARE complex. The possible occurrence of a physical interaction of mGluR4 with Munc-18-1 as well as with t-SNAREs (syntaxin 1, SNAP25) and the calcium sensor synaptotagmin-2 is further supported by the results of a proteomics study (*15*). Furthermore, there is evidence suggesting that the interaction between mGluR4 and Munc-18-1 might be modulated by Ca^2+^, partially via calmodulin (*57*). Although further studies will be required to further investigate and clarify the functional role of these interactions, our super-resolution microscopy results together with previous biochemical and functional data point to a high level of spatial and functional integration between mGluR4s, Ca_V_2.1 channels, and the machinery responsible for vesicle docking and fusion.

Overall, our data reveal a previously unknown high spatial organization of mGluR4s at presynaptic AZs in the mouse cerebellum. This provides a new important ultrastructural basis to understand how these prototypical presynaptic GPCRs modulate neurotransmitter release.

## MATERIALS AND METHODS

### Animals

Wild-type adult FVB mice were used for the preparation of the mouse cerebellar slices. All animal work was done according to regulations of the relevant authority, the government of Lower Franconia, Bavaria.

### Antibodies and reagents

The anti-mGluR4a guinea pig polyclonal antibody (K44) was provided by R. Shigemoto (Institute of Science and Technology, Austria). It was generated against a synthetic peptide corresponding to the C terminus of rat mGluR4 (amino acid residues 890 to 912). Specificity of the antibody was verified by immunoblot analysis of membrane fractions from rat hippocampus and from CHO cells transfected with mGluR4a, mGluR7a, mGluR7b, or mGluR8 complementary DNA (cDNA) (*17*, *35*). The rabbit polyclonal antibody against the α1 subunit of Ca_V_2.1 (catalog no. 152203), rabbit polyclonal antibody against bassoon (catalog no. 141013), and monoclonal mouse antibody against Munc-18-1 (catalog no. 116011) were from Synaptic Systems (Göttingen, Germany). The mouse monoclonal antibody against bassoon (catalog no. SAP7F407) was from Enzo Life Sciences (NY, USA). TetraSpeck microspheres (0.1 μm), Alexa Fluor 647–conjugated goat anti-guinea pig (catalog no. A21450), Alexa Fluor 647–conjugated goat anti-rabbit (catalog no. A21245), Alexa Fluor 532–conjugated goat anti-mouse (catalog no. A11002), and Alexa Fluor 532–conjugated goat anti-rabbit (catalog no. A11009) secondary polyclonal antibodies were from Thermo Fisher Scientific (Waltham, MA, USA). SNAP-Surface Alexa Fluor 647 was from New England Biolabs (Ipswich, MA, USA). Cell culture reagents and Lipofectamine 2000 were from Thermo Fisher Scientific (Waltham, MA, USA). Fetal bovine serum was from Biochrom (Berlin, Germany). Normal goat serum was from Sigma-Aldrich (Steinheim, Germany). All other chemicals and reagents were from AppliChem (Darmstadt, Germany).

### Plasmids and cloning

A plasmid coding for mGluR4 with a hemagglutinin (HA) tag and a fast labeling variant of the SNAP-tag (SNAPf) (*58*) fused to its N terminus was generated by replacing the sequence of the γ-aminobutyric acid type B receptor subunit 1a (GABAB_1a_) with wild-type mouse mGluR4 cDNA and the SNAP-tag (*59*) with the SNAPf tag in a previously described construct (provided by J. P. Pin, Institut de Génomique Fonctionnelle, Montpellier, France) (*26*). The construct was correctly expressed on the plasma membrane and was functional in adenosine 3′,5′-cyclic monophosphate (cAMP) assays. Plasmids expressing CD86 with either one or two SNAP-tags fused to its N terminus were described previously (*33*).

### Cell culture and transfection

CHO cells were from the American Type Culture Collection. Cells were cultured in phenol red–free Dulbecco’s modified Eagle medium/F-12 supplemented with 10% fetal bovine serum, penicillin (100 IU/ml), and streptomycin (0.1 mg/ml) at 37°C, 5% (v/v) CO_2_. The cells were mycoplasma-free as verified by polymerase chain reaction. Coverslips for single-molecule microscopy were extensively cleaned as described previously (*33*). Cells (250,000) were seeded onto clean 24-mm round glass coverslips placed in six-well plates and allowed to adhere overnight. CHO cells were then transfected with Lipofectamine 2000 according to the manufacturer’s protocol. For each well, 2 μg of DNA and 6 μl of Lipofectamine 2000 were used. Cells were labeled and imaged 4 to 5 hours after transfection as previously described (*33*).

### Live-cell labeling of SNAP-tagged constructs

CHO cells expressing SNAP-tagged membrane receptors were washed twice with phosphate-buffered saline (PBS). Covalent labeling of the SNAP-tag was conducted by incubating the cells for 20 min at 37°C, 5% (v/v) CO_2_ with 2 μM of the membrane-impermeable SNAP substrate SNAP-Surface Alexa Fluor 647 diluted in complete phenol red–free culture medium. At the end of the incubation, the cells were washed three times with complete phenol red–free medium, with 5-min incubation at 37°C, 5% (v/v) CO_2_ after each wash. Last, the cells were washed twice with PBS for 5 min at room temperature (RT) and fixed with 4% paraformaldehyde (PFA) for 15 min at RT.

### Cerebellar slice preparation

Euthanized mice were transcardially perfused with 0.2 M sodium phosphate buffer (PB) containing heparin (10 IU/ml), followed by 4% PFA in PB (pH 7.4) for 10 min. Brains were isolated and postfixed with 4% PFA in PB overnight at 4°C. Samples were then transferred to 30% sucrose in PBS for approximately 24 hours. The next day, the cerebella were dissected from the rest of the brain, included in Tissue-Tek O.C.T. (Sakura Finetek, Alphen aan den Rijn, The Netherlands), and snap-frozen in isopentane precooled with liquid nitrogen. Samples were then sliced into thin sections of 1.5 μm on a Leica CM3050S cryostat. The slices were collected on silanized 18-mm round coverslips and stored at −80°C.

### Immunofluorescence staining of cerebellar slices

Cerebellar slices were incubated with 0.02 M glycine in PBS to quench aldehyde groups. Samples were then blocked and permeabilized by incubation with a blocking solution consisting of 1% bovine serum albumin, 5% normal goat serum, and 0.3% Triton X-100 in PBS for 2 hours at RT. Subsequently, the samples were incubated with the appropriate concentrations of primary antibodies in the blocking solution overnight at 4°C. Primary antibodies were used in the following dilutions: guinea pig anti-mGluR4 (1:100 to 1:200; stock solution concentration = 0.94 mg/ml), mouse anti-bassoon (1:200 to 1:400), rabbit anti-bassoon (1:200 to 1:400), rabbit anti-Ca_V_2.1 (1:200), and mouse anti–Munc-18-1 (1:400). In case of double labeling, slices were simultaneously incubated with both primary antibodies. At the end of the incubation, the slices were washed twice for 10 min with the blocking solution, followed by two washing steps of 40 min each with the blocking solution. Sections were incubated with the Alexa Fluor–conjugated secondary antibodies diluted at 1:200 in blocking solution for 2 hours at RT. The slices were washed twice for 10 min with the blocking solution, followed by two washing steps for 40 min with the blocking solution. The samples were kept in PBS at 4°C until imaging.

### Immunofluorescence staining of transfected cells

Cells were washed twice with PBS for 5 min each and fixed with 4% PFA for 15 min at RT. Cells were then washed four times with PBS for 5 min each. Immunofluorescence was carried out as described for cerebellar slices.

### *d*STORM imaging

Samples were imaged in the presence of a reducing agent to enable reversible photoswitching of the fluorophores. The imaging buffer consisted of 100 mM β-mercaptoethylamine (Sigma-Aldrich, Steinheim, Germany) in PBS (pH 7.4 to 7.8), as previously described (*32*). Image acquisition was performed on an inverted fluorescence wide-field setup custom-built around an Olympus IX-71 microscope equipped with an oil-immersion objective [APON 60×; numerical aperture (NA), 1.49; Olympus, Tokyo, Japan], a nose-piece stage (IX2-NPS, Olympus) to reduce stage vibration and drift, 514-nm (500 mW) and 639-nm (1000 mW) solid-state lasers (OPSL, Genesis MX STM-Series, Coherent, Santa Clara, CA, USA), and a suitable dichroic mirror (R442/514/635, Chroma, Bellows Falls, Vermont, USA). The fluorescence emission of Alexa Fluor 647 and Alexa Fluor 532 dyes was acquired sequentially and projected on two separate electron-multiplying charge-coupled device (EMCCD) cameras (iXon Ultra 897, Andor, Belfast, Northern Ireland) by means of a dichroic mirror (630 DCXR customized, Chroma) and two bandpass filters (582/75 and 679/41 BrightLine series, Semrock, Rochester, NY, USA). The excitation intensity ranged from 1 to 5 kW/cm^2^, depending on the fluorophore and labeling density. For each color, 40,000 frames were acquired at 60 Hz. This single-molecule localization data were analyzed using the open source software rapidSTORM (version 3.3). The fitting process and the reconstruction of super-resolved images were performed as previously described (*60*). Localization precision was estimated on the basis of a nearest neighborhood analysis approach (see Supplementary Results for details) (*61*).

### Cluster analysis

Cluster analysis was performed using home-written algorithms in Mathematica 11.1. To gain a global assessment of the molecular distribution, we used the normalized Ripley’s *K* function (Ripley’s *H* function) (*39*). A region of 32.9 μm × 32.9 μm in the *d*STORM image was examined. Positive values of *H*(*r*) are indicative of clustering, while negative values indicate dispersion. The *r* value at which *H*(*r*) is maximal is a crude indicator of the domain radius. For comparison, we used simulated localizations following a Neyman-Scott distribution, consisting of a random number of daughter events following a Poisson distribution, centered around random uniformly distributed parent events. Each parent event had, on average, 20 daughter events. The coordinates of the daughter events were distributed as a two-dimensional Gaussian around each parent event with SD of 20 nm. In addition, we used simulated localizations with a random uniform distribution. Nanoclusters were identified using the DBSCAN algorithm (*36*). Briefly, this hierarchical clustering algorithm computes for every localization in the dataset the number of localizations within distance *r*. If this number is equal or higher than a defined threshold (minPts), then those localizations are assigned to the same initial cluster. For each localization in the initial cluster, the algorithm then searches for localizations within distance *r*. If the number of localizations is equal or higher than minPts, then the boundary of the cluster is expanded to include the new localization. The expansion terminates, and the boundary of the cluster is defined once all localizations have been considered.

### Changes of mGluR4 density with distance from AZ border

AZs were recognized on the basis of the presence of bassoon clusters, identified using the DBSCAN algorithm (*r* = 80 nm, 20 minPts). Clusters with surface area of 100,000 to 600,000 nm^2^, corresponding to well-developed AZs (diameter, ~350 to 860 nm), were selected. The orientation of each AZ was then estimated by computing its inertia moment eccentricity (IME) and bounding box elongation (BBE). IME was defined as $\sqrt{1-M2/M1}$, where M1 and M2 correspond to the short and long principal axes, respectively, calculated from the inertia moment vector. BBE was defined as 1 minus the ratio of the length of the short to that of the long axis of the smallest oriented bounding box containing the localizations of a cluster. Both indexes can vary between 0 and 1, where 0 indicates a circular shape and 1 indicates a completely elongated one. En face AZs were identified as those with IME values between 0 and 0.9 and BBE values between 0 and 0.5. Thereafter, the distances between mGluR4 localizations and the border of each AZ were analyzed. The analysis was extended to 20 nm outside the border to ensure that localizations lying close to it were also included. Average densities were calculated both inside and outside AZs. In the latter case, the area between the AZ border and an orthogonal distance of 2000 nm from every border point was used.

### Estimation of mGluR4 numbers

To estimate the number of mGluR4s residing within individual nanoclusters, the primary antibody concentration required to saturate the mGluR4 epitope had to be identified. For this purpose, a titration curve was constructed by staining cerebellar slices with increasing primary antibody concentrations (1:50, 1:100, 1:200, 1:400, 1:2000, and 1:20,000). The concentration of the secondary antibody (Alexa Fluor 647–conjugated anti-guinea pig antibody) was constant for all experiments (1:200 dilution). Data were fitted to a logistic function

$$y=N_{\text{NC},\text{max}}^{\text{mGluR}4}+\frac{N_{\text{NC},\text{min}}^{\text{mGluR}4}-N_{\text{NC},\text{max}}^{\text{mGluR}4}}{1+{\left(x\backslash x_{0}\right)}^{p}}$$

where *y* is the average number of localizations per nanocluster, $N_{\text{NC},\text{max}}^{\text{mGluR}4}$ corresponds to the average number of localizations per nanocluster under saturating conditions, $N_{\text{NC},\text{min}}^{\text{mGluR}4}$ corresponds to the average number of localizations per nanocluster under limiting dilution (i.e., the average number of localizations per each primary antibody), *x* corresponds to the primary antibody concentration, *x*_0_ is the primary antibody concentration at the sigmoid’s midpoint, and *p* is the Hill coefficient. A saturating dilution of 1:100 was used in subsequent experiments.

### Distance-based colocalization analysis

The distance-dependent colocalization between localizations into two separate channels (*A* and *B*) was calculated on the basis of a modification of our previously described method (*34*). Briefly, the localizations in both channels were first binned to produce super-resolved images with 5 × 5–nm pixel size. A Gaussian filter with increasing SD was then applied to produce images of channel *B* with progressively lower resolution, which we used to probe the colocalization between channel *A* and *B* at increasing distances. For each considered distance, a colocalization index (*I*) was calculated on the basis of the following equation

$$I=\frac{(\langle B_{\text{loc}}\rangle -\langle B\rangle )\langle A\rangle }{\langle B\rangle (\langle A_{\text{loc}}\rangle -\langle A\rangle )}$$

defined for 〈*B*_loc_〉 ≥ 〈*B*〉, where 〈*A*_loc_〉 and 〈*B*_loc_〉 are the averages of the interpolated intensities of the two channels at each localization in *A*, and 〈*A*〉 and 〈*B*〉 are the average intensities of the two channels. The colocalization index *I* can assume values between 0, in case of lack of correlation between the two channels, and 1 in case of perfect correlation. Areas with no or low localization density in channel *A* were excluded from the analysis. Results were compared to those obtained with an equal number of random uniformly distributed localizations or a comparable number of random localizations following a Neyman-Scott process.

### NN analysis of cluster centroids

Localization clusters were identified with the DBSCAN algorithm. Subsequently, the NN distances between the cluster centroids of the first (P1) and second (P2) population were estimated. As a control, the centroid positions of the second population were randomized, and the NN distances between P1 and the randomized P2 were computed. The resulting histogram of NN distances for the randomly distributed data was normalized so that the number of localizations at long distances (>250 nm) was equal to that measured between P1 and P2. Last, a distribution compensated for the random component was calculated by subtracting from the distribution of the NN analysis the normalized one obtained with randomized P2.

### Single-molecule microscopy

Single-molecule microscopy was performed using TIRF illumination on a custom Nikon Eclipse Ti TIRF microscope equipped with 405-, 488-, 561-, and 640-nm diode lasers (Coherent), a quadruple band excitation filter, a 100× oil-immersion objective (CFI Apo TIRF 100×; NA, 1.49), two beam splitters, four separate EMCCD cameras (iXon DU897, Andor), hardware focus stabilization, and a temperature control system. Coverslips were mounted in a microscopy chamber filled with imaging buffer. The objective and the sample were maintained at 20°C by means of a water-cooled inset and an objective ring connected to a thermostat-controlled water bath. Image sequences (400 frames) were acquired in crop and frame-transfer mode, resulting in an acquisition speed of 35 frames/s. Single particle detection and tracking were performed using the u-track algorithm (*62*) in MATLAB as previously described (*33*).

### Single-molecule intensity distribution analysis

The number of receptors per particle in single-molecule image sequences was estimated as previously described (*33*). Briefly, for each particle, the intensities from the beginning of the sequence to the first stepwise change in fluorescence and up to 20 frames were averaged. The distribution of the particle intensities was then fitted with a mixed Gaussian model, according to the following equation

$$\varphi (i)=\sum_{n=1}^{n\;\text{max}}A_{n}\frac{1}{n\sigma \sqrt{2\pi }}e^{-\frac{{\left(i-n\mu \right)}^{2}}{2{\left(n\sigma \right)}^{2}}}$$

where φ (*i*) is the frequency of particles with intensity *i*, *n* is the component number, *A_n_* is the area under the curve of each component, and μ and σ are the mean and SD of the intensity of the used fluorophore, respectively. The intensity distribution of monomeric receptors (Alexa Fluor 647–labeled SNAP-CD86) was used as an initial estimate of μ and σ. However, because minor differences in particle intensities among different image sequences can occur, μ and σ were finely adjusted for each individual image sequence based on the particle intensities of the last 60 frames, when a large fraction of fluorophores is photobleached and a predominant peak corresponding to the intensity of single fluorophores is present. The relative abundance of each individual component was then calculated from the corresponding areas under the curve (*A_n_*).

### Statistics

Data are reported as means ± SEM, unless otherwise indicated. Statistical analyses were conducted using Prism 6 software (GraphPad Software, La Jolla, CA, USA). Two-sided paired *t* test was used to assess differences between two groups. Analysis of variance (ANOVA) was used to assess differences between three or more groups, followed by Holm-Sidak’s test. Differences were considered significant for *P* < 0.05.

## Supplementary Material

aay7193_SM.pdf

## SUPPLEMENTARY MATERIALS

Supplementary material for this article is available at http://advances.sciencemag.org/cgi/content/full/6/16/eaay7193/DC1

## Appendix

View/request a protocol for this paper from *Bio-protocol*.
