# Supplementary material for: Super-resolution imaging reveals the nanoscale organization of metabotropic glutamate receptors at presynaptic active zones
Source: Sci Adv. 2020 Apr 15;6(16):eaay7193. doi: 10.1126/sciadv.aay7193 (PMC7159906; doi:10.1126/sciadv.aay7193)
Supplement: aay7193_SM.pdf [file aay7193_SM.pdf]

Science Advances
AAAS

advances.sciencemag.org/cgi/content/full/6/16/eaay7193/DC1

Supplementary Materials for

Super-resolution imaging reveals the nanoscale organization of metabotropic glutamate receptors at presynaptic active zones

Sana Siddig, Sarah Aufmkolk, Sören Doose, Marie-Lise Jobin, Christian Werner, Markus Sauer*, Davide Calebiro*

*Corresponding author. Email: m.sauer@uni-wuerzburg.de (M.S.); d.calebiro@bham.ac.uk (D.C.)

Published 15 April 2020, *Sci. Adv.* **6**, eaay7193 (2020)
DOI: 10.1126/sciadv.aay7193

This PDF file includes:

Supplementary Results
Figs. S1 to S10

Supplementary Results

Precision and accuracy of two-color *d*STORM

The localization precision of single-molecule localization microscopy is indirectly proportional to the square of the number of detected photons per on-state fluorophore (*42*). Therefore, the quality of the data depends highly on the chosen fluorophore. To analyze the average experimental precision, we used a nearest neighbor analysis-based approach that takes into account also the stability of the setup and the accuracy of the employed fitting algorithm (*61*).

An important factor contributing to the accuracy of two-color data is the quality of the alignment of the two emission channels, which can have a larger impact than the localization error. To limit this potentially important source of error, we simultaneously acquired images of beads stained with multiple fluorophores (Tetraspeck^{TM}) on both cameras. We then used these data to generate a registration matrix, which we applied to register the images between the two channels. This allowed us to also correct for chromatic aberrations and slight differences in detection pathways between the two channels. The registration was performed with the Fiji plugin bUnwarpJ and the obtained pixel-to-pixel transformation was used directly in rapidSTORM to create corrected localization lists. The accuracy of the two-color registration was tested over several hours and with multiple bead samples. From these analyses we estimated an interchannel localization precision of ~ 20 nm.

Estimation of number of epitopes recognized by the mGluR4 antibody.

The number of epitopes recognized by the mGluR4 antibody was estimated by performing single-molecule TIRF microscopy imaging in fixed CHO cells transiently transfected with mGluR4 for 4h to achieve low physiological expression levels. To calculate the number of epitopes per receptor we compared the intensity distributions of the fluorescent particles detected under saturating conditions (1:100) vs. limiting dilution (1:10^6) of the anti-mGluR4 primary antibody. The resulting distributions were fitted with a mixed Gaussian model as previously described (*33*). The experiments gave complex distributions (see **Fig. 3E**), which can be explained by a combination of three factors: the binding of more than one secondary antibody to each primary antibody; a

variable number of fluorophores carried by each secondary antibody and the underlying supramolecular organization of mGluR4. To compare the two distributions, we considered their first peaks. Under conditions of limiting dilution of the primary antibody, this peak should correspond to one primary antibody bound to the smallest complex present and recognized by secondary antibodies with the lowest degree of labeling (i.e. carrying the lowest number of fluorophores). In the presence of saturating concentrations of the primary antibody, the first peak should correspond to n x m primary antibodies, each bound to secondary antibodies with the lowest degree of labeling, where n is the number of receptors per cluster and m is the number of primary antibodies bound to each receptor. Thus, the number of primary antibodies bound to each receptor can be estimated by dividing the intensity of the first peak under saturating conditions by the one under limiting dilution conditions, which gave a value of 1.8 ± 0.22, and further dividing this number by the smallest size of receptor complexes present, which in the case of mGluR4 was 2, as estimated by single-molecule TIRF microscopy. From these calculations, we estimated that no more than one anti-mGluR4 antibody was capable to simultaneously bind to one mGluR4.

Supplementary Figures

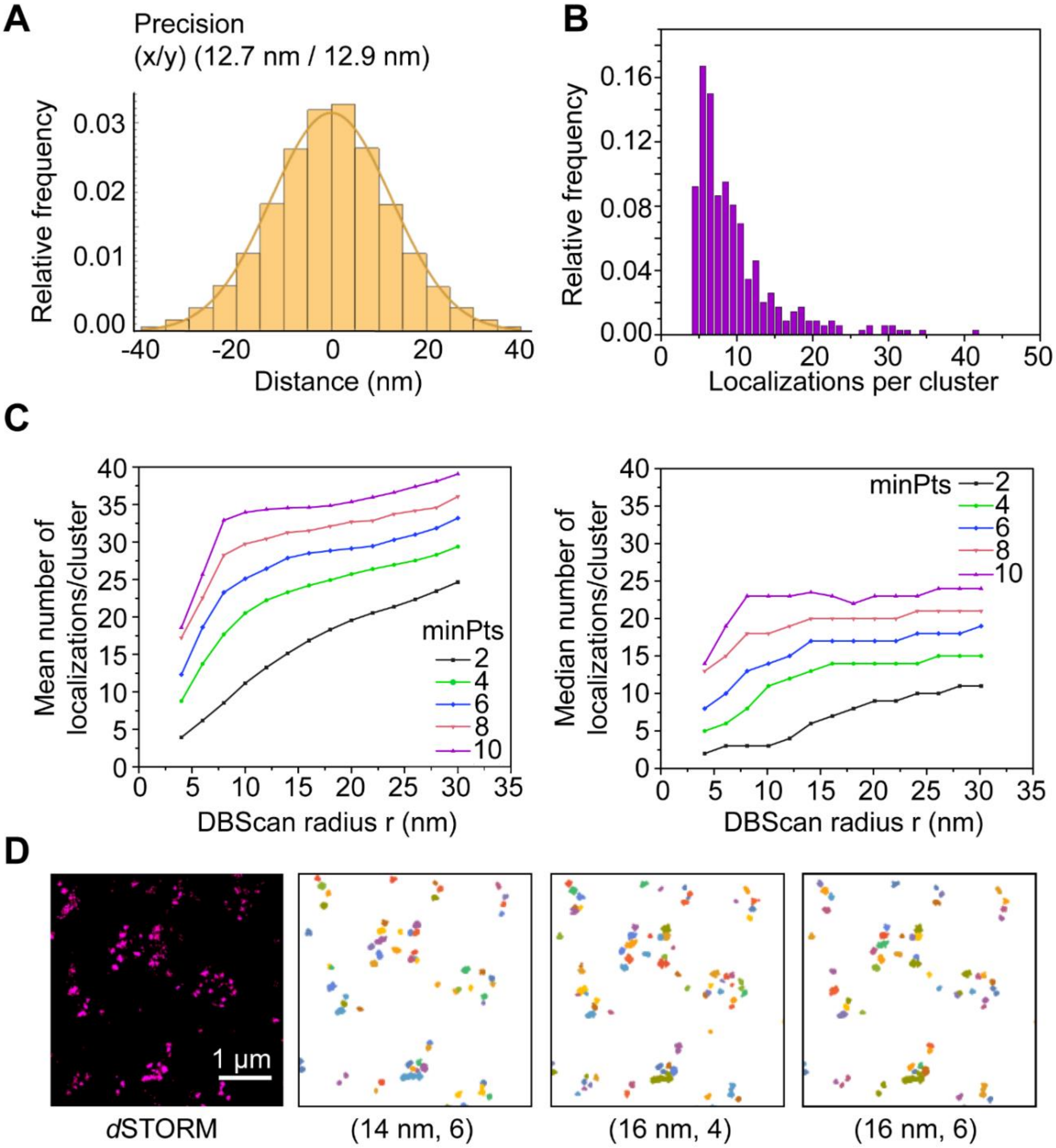

Figure S1. Selection of parameters for DBSCAN analysis of mGluR4 localizations. (**A**) Determination of the localization precision (standard deviation) from localization data of Alexa Fluor 647 labeled mGluR4s on a cerebellar section. The analysis gave a value of ~ 13 nm in the lateral direction. This value was used as a guide for selection of the searching radius for the DBSCAN analysis. (**B**) Histogram of the frequency of localizations per cluster in the case of non-specifically adsorbed Alexa Fluor 647-conjugated secondary antibodies (applying DBSCAN with r = 16 nm, minPts = 4). (**C**) Parameter scan for the DBSCAN analysis. The mean (left) and the median (right) number of localizations belonging to isolated fluorophore-labeled mGluR4 are

plotted over the DBSCAN radius (r) using different values for the minPts parameter. The presence of a plateau indicates the correct identification of clusters with the chosen set of parameters. (**D**) Visual representation of the clusters identified by the DBSCAN analysis with different sets of parameters. Each cluster is identified by a different color. Values of r = 16 nm and minPts = 4 were selected and used for subsequent analyses.

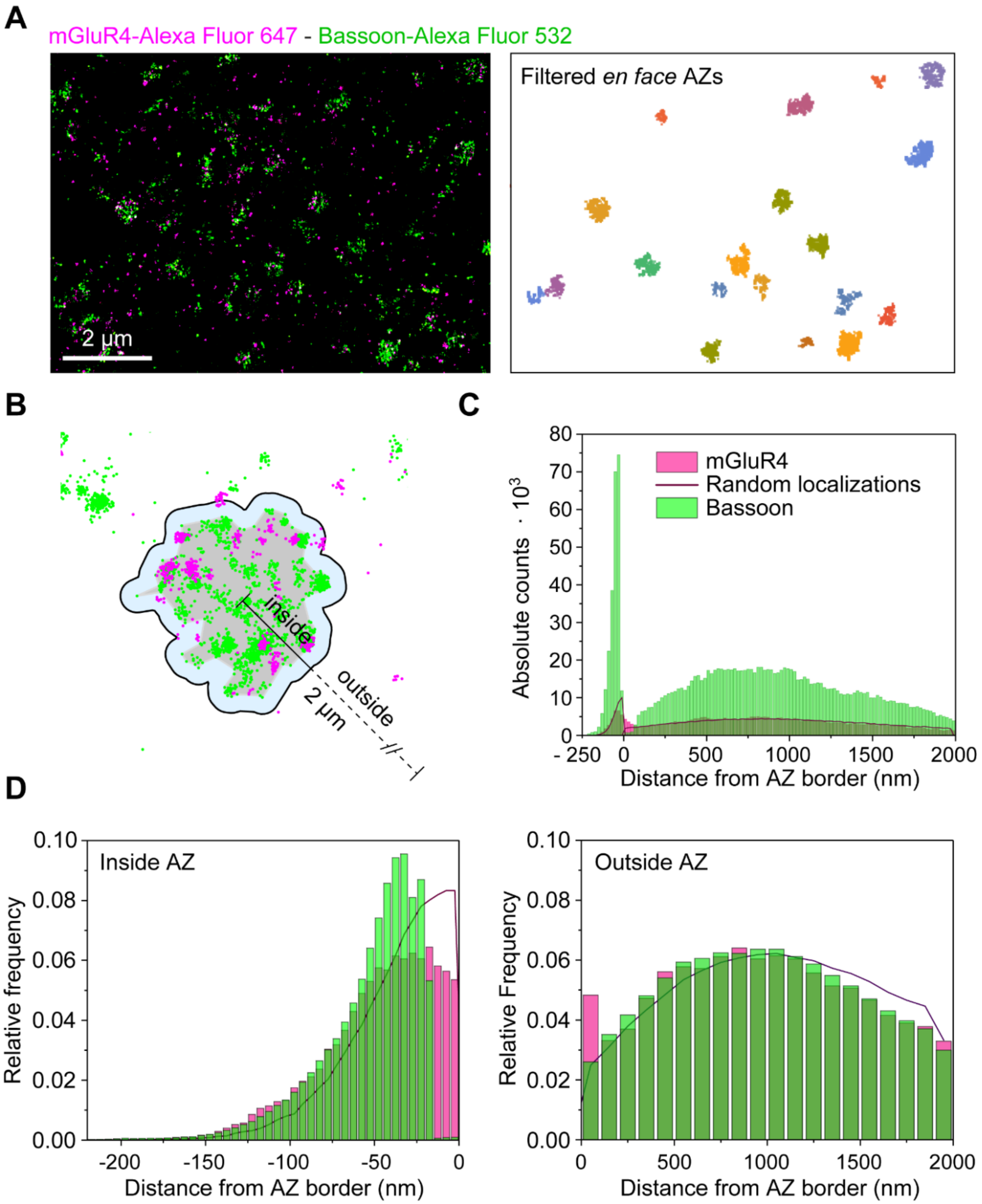

Figure S2. mGluR4 distribution within parallel fiber AZs. (**A**) Identification of *en face* view AZs**.** Left, Representative *d*STORM image of mGluR4 (magenta) and bassoon (green). Right, *en face* AZs in **A**, identified by applying DBSCAN (r = 80 nm, 20 minPts) followed by filtering based on their surface, inertia moment eccentricity and bounding box elongation (see Methods for details). (**B-D**) Distribution of mGluR4 localizations with respect to their distances from the AZ

border. (**B**) Principle of the analysis. Shown are the mGluR4 and bassoon localizations within a representative *en face* AZ. The AZ border was set at a distance of 20 nm outside the boundary defined by the bassoon localizations (see Methods for details). (**C**) Results of the analysis. Shown are histograms of the absolute number of mGluR4 and bassoon localizations as a function of their distance from the AZ border. Negative and positive values correspond to distances inside and outside the AZ border, respectively. Results obtained using a number of random localizations equal to that of mGluR4s are given for comparison. (**D**) Histograms reporting the relative frequencies of mGluR4 and bassoon localizations as a function of their distance from the AZ border. Left, inside AZ. Right, outside AZ. The mGluR4 distribution was overall similar to that of bassoon and to the simulated random localization distribution (solid line) indicating a rather homogenous mGluR4 distribution within the AZ for the ensemble of all analyzed AZs.

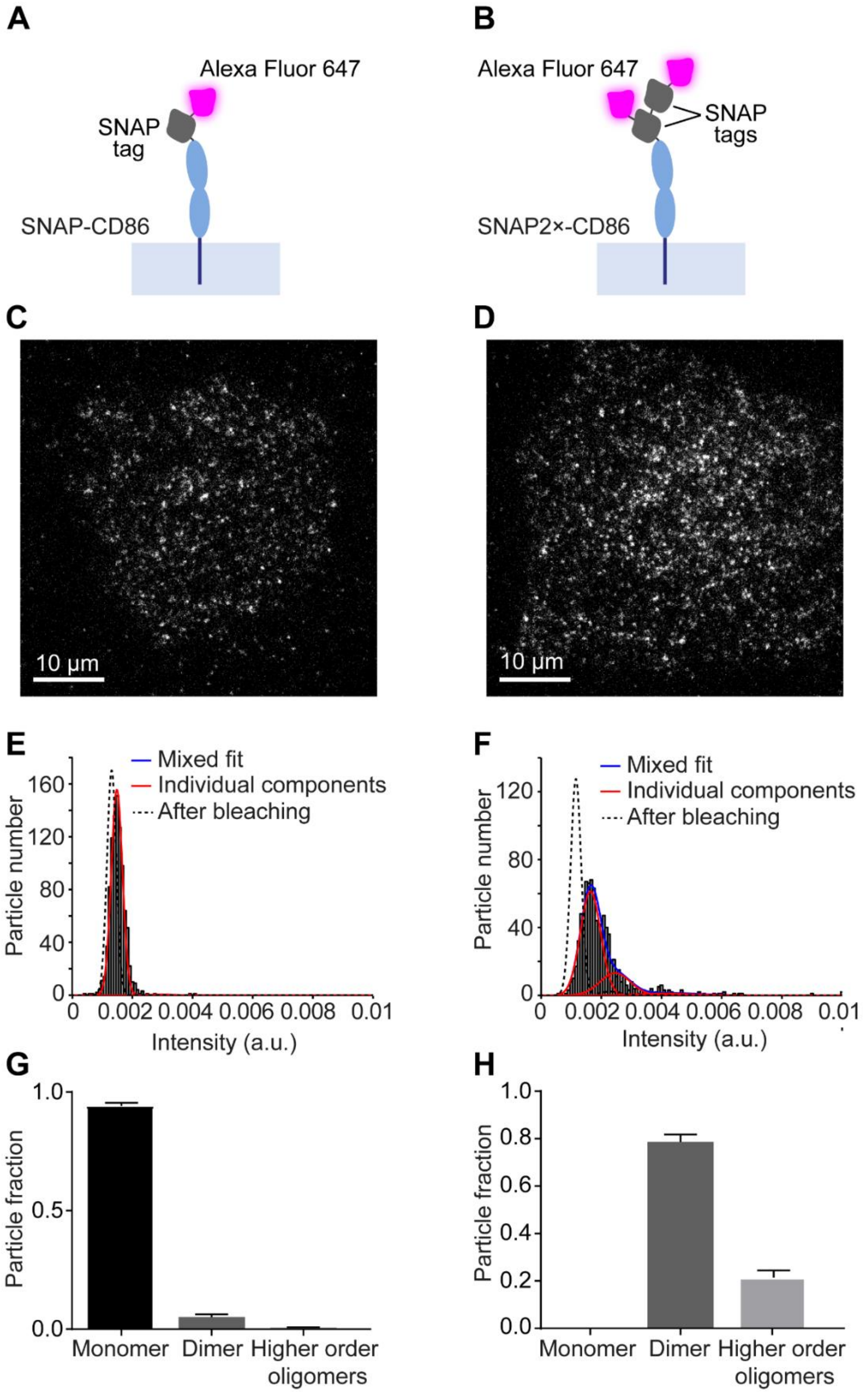

Figure S3. Validation of the single-molecule method to quantify the size of receptor complexes. (**A-D**) Representative results. CHO cells were transiently transfected with CD86 constructs carrying either one (SNAP-CD86) (**A**) or two (SNAP2×-CD86) (**B**) SNAP-tags at their N-terminus and labelled at 1:1 stoichiometry with a saturating concentration (2 µM) of an Alexa

Fluor 647 benzylguanine derivative. Cells expressing SNAP-CD86 (**C**) or SNAP2×-CD86 (**D**) were then fixed and imaged by TIRF microscopy. SNAP2×-CD86 was used as dimeric control. Cells expressing SNAP-CD86 and SNAP2×-CD86 at low physiological densities, corresponding to 0.29 ± 0.07 and 0.40 ± 0.1 (s.d.) fluorescently labeled CD86s/μm^2, respectively, were used for subsequent analyses. Dots in **C** and **D** represent individual receptor particles, which were identified with an automated single particle detection algorithm. (**E,F**) Histograms reporting the distributions the intensities of the particles obtained from representative SNAP-CD86 (**E**) and SNAP2×-CD86 (**F**) image sequences. The results obtained considering the last frames of each image sequence, when most fluorophores are bleached (dashed line), were used as internal reference for the distribution expected for monomeric receptors. (**G,H**) Relative abundance of monomers, dimers and higher order oligomers/nanoclusters estimated by fitting intensity distributions as in **E** or **F** with a mixed Gaussian model (see Methods for details). Data are mean ± s.e.m. of 14 (10,290) and 13 (12,415) cells**,** respectively (number of particles in brackets).

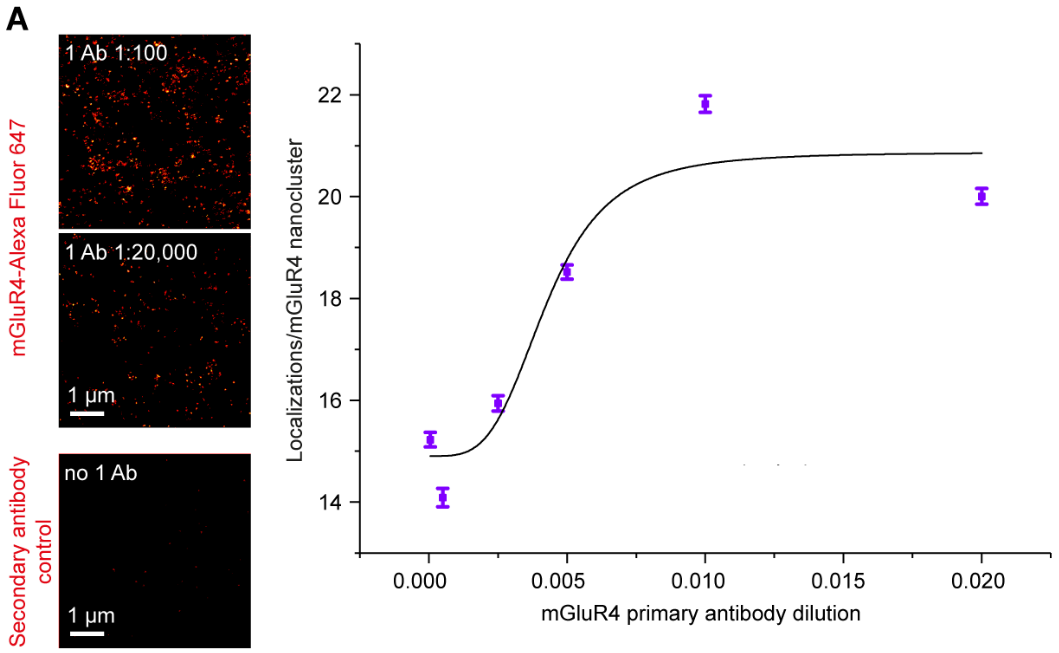

B

	mGluR4-Alexa Fluor 647 1:100	mGluR4-Alexa Fluor 647 1:20,000	Secondary antibody (SAb) control	Ratio SAb control/ mGluR4 1:100	Ratio SAb control/ mGluR4 1:20,000
Localizations/Field of view	$89.6 \pm 9.9 \cdot 10^4$	$6.81 \pm 0.65 \cdot 10^4$	$0.22 \pm 0.03 \cdot 10^4$	0.002	0.03
Localizations/µm²	841 ± 93	62.9 ± 6.0	2.05 ± 0.65		
Nanoclusters/Field of view	19018 ± 1744	3198.5 ± 264.2	83 ± 15	0.004	0.02
Nanoclusters/µm²	17.9 ± 1.64	2.9 ± 0.2	0.08 ± 0.01		

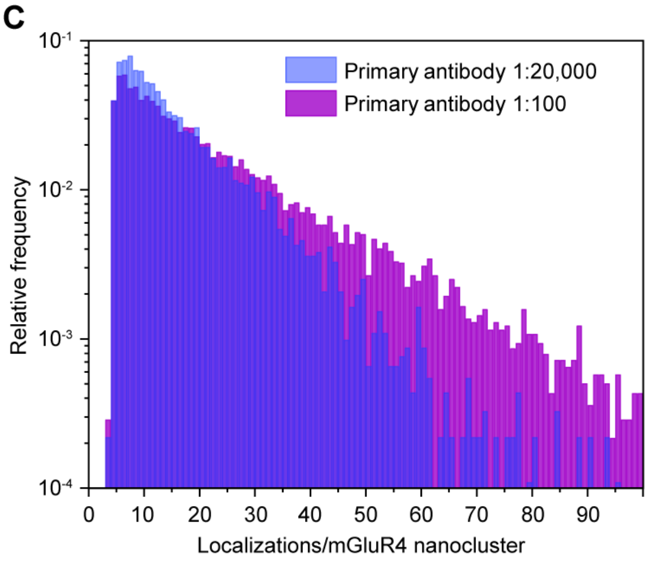

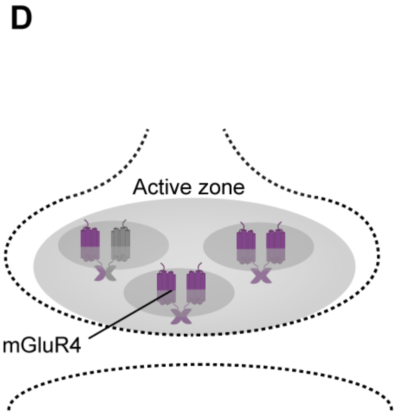

Figure S4. Stoichiometry of mGluR4 complexes at parallel fiber AZs. (**A**) Titration curve of the primary antibody against mGluR4 employed in this study. Left, representative *d*STORM images of the mouse cerebellum stained with either a saturating concentration (top) or a limiting dilution of the primary antibody (1 Ab) against mGluR4a, or omitting the primary antibody (bottom). Right, graph showing the dependency of the number of localizations detected per

nanocluster on the concentration of the primary antibody. n = 15,097, 13,974, 12,264, 6,532, 3,548, 6,399 nanoclusters. Data were fitted with a logistic function. (**B**) Summary of the number of localizations and nanoclusters detected in the three conditions reported in **A**. (**C**) Distribution of the number of localizations per nanocluster measured with either a limiting dilution or saturating concentration of the primary antibody. n = 6,399 and 13,974 nanoclusters, respectively. (**D**) Schematic view of the results, showing the organization of mGluR4 in nanodomains (dark gray circles), containing on average 1–2 detected mGluR4 subunits.

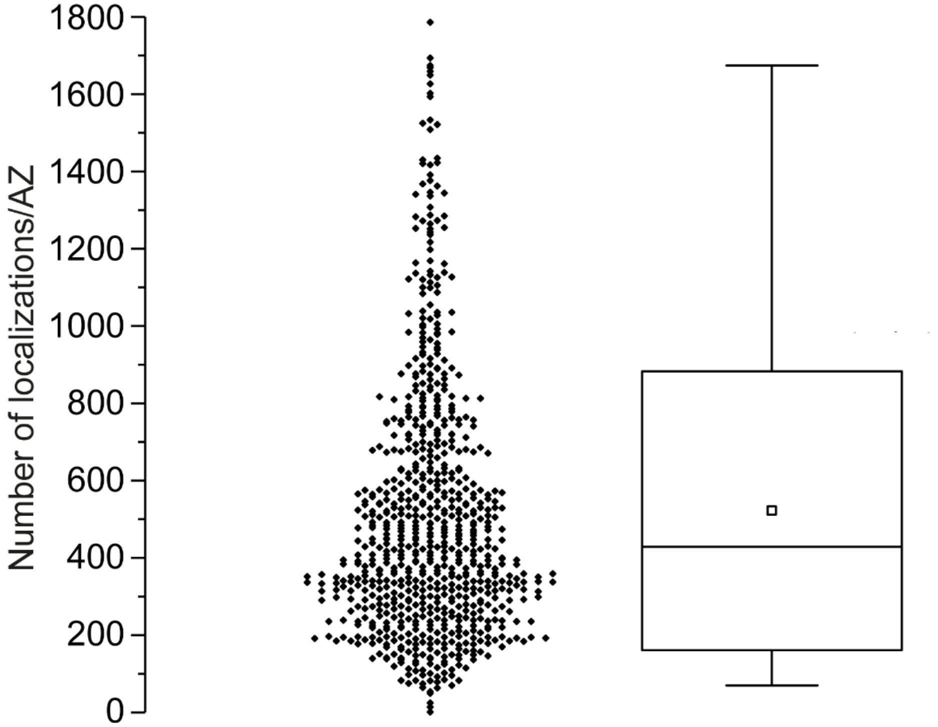

Figure S5. Quantification of number of mGluR4s per parallel fiber AZ. The results are shown both as beeswarm plot (left) and boxplot (right). The boxplot reports the mean (square; 522.1 localizations, i.e. ~ 35 receptors), median (429 localizations, i.e. ~ 29 receptors), 25% percentile (276.5 localizations, i.e. ~ 19 receptors) and 75% percentile (678 localizations, i.e. ~ 46 receptors). n = 699.

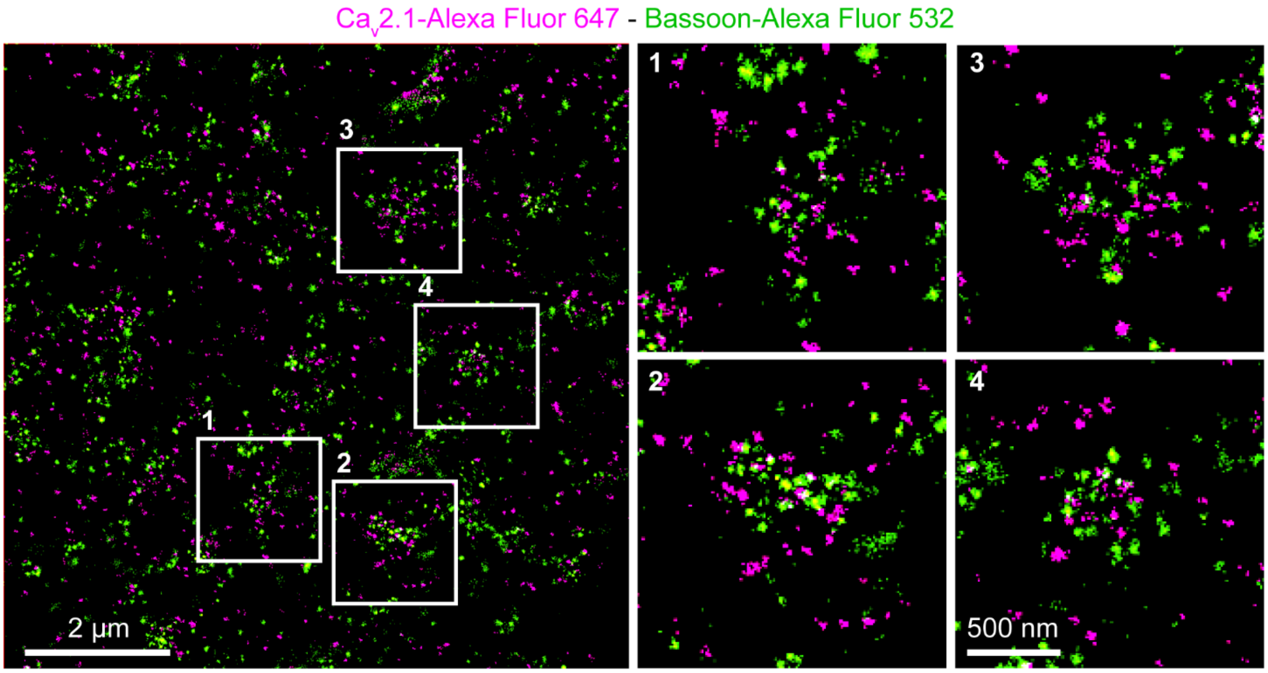

Figure S6. Two-color super-resolution (*d*STORM) imaging of Ca$_V$2.1 channels and bassoon. Left, representative two-color *d*STORM image revealing the organization of Ca$_V$2.1 channels (magenta) relative to bassoon (green). Right, enlarged views corresponding to the regions delimited by the white boxes.

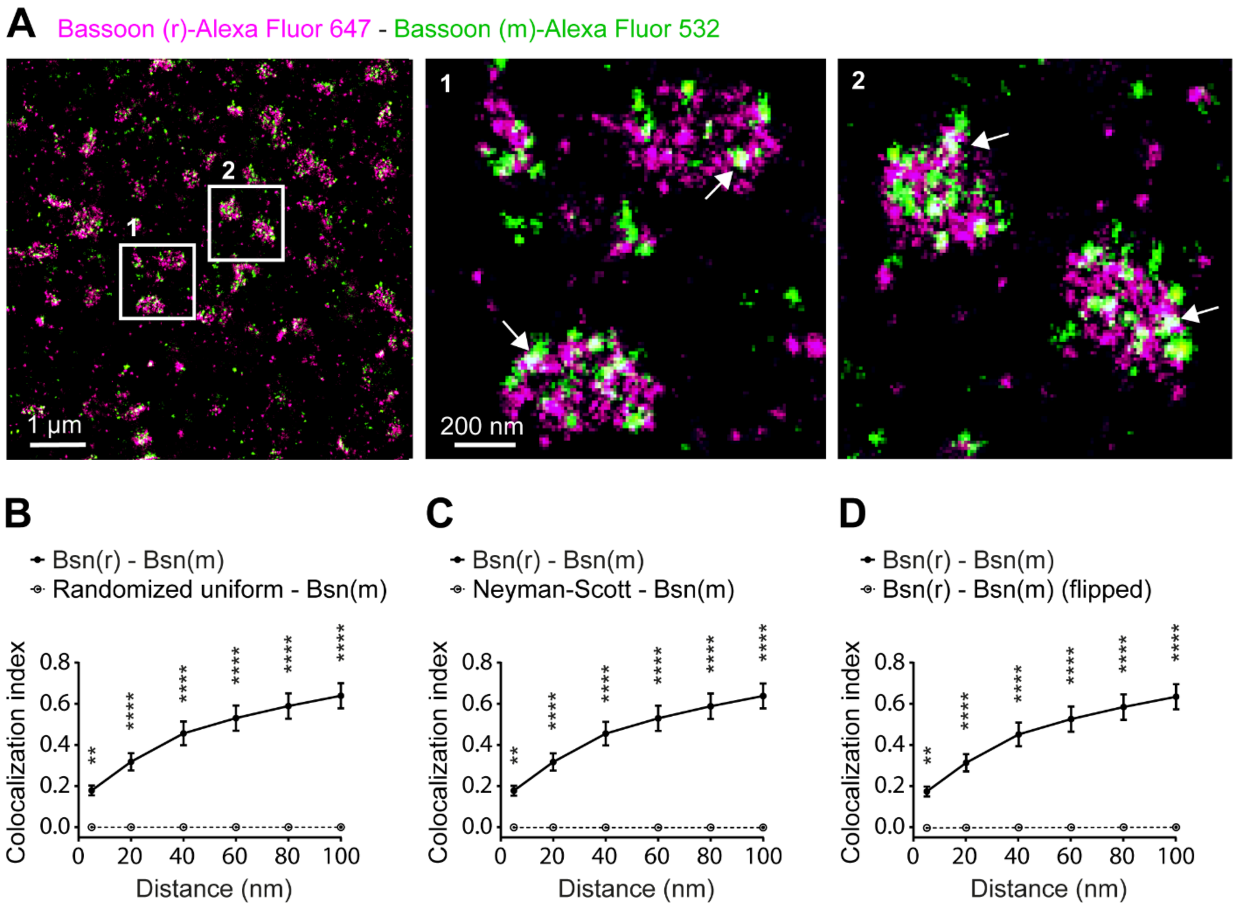

Figure S7. Validation of the distance-based colocalization analysis. (**A**) Two-color *d*STORM imaging of cerebellar slices simultaneously stained with two distinct primary antibodies against bassoon (Bsn) raised in rabbit (r) and mouse (m). Left, representative *d*STORM image. Middle and right, enlarged views corresponding to the regions delimited by the white boxes. White (arrows) indicates perfect colocalization. (**B-D**) Corresponding distance-based colocalization analysis. Shown are colocalization index values calculated over increasing distances. Results were compared to those obtained by replacing Bsn(r) localizations with either an equal number of random uniformly distributed localizations (**B**), or a comparable number of localizations following a Neyman-Scott distribution (**C**), or by horizontally flipping the Bsn(m) channel (**D**). Data are mean ± s.e.m. of 3 *d*STORM images. Differences are statistically significant by two-way ANOVA followed by Holm-Sidak's test. $^{**}P < 0.01$ and $^{****}P < 0.0001$ versus corresponding control.

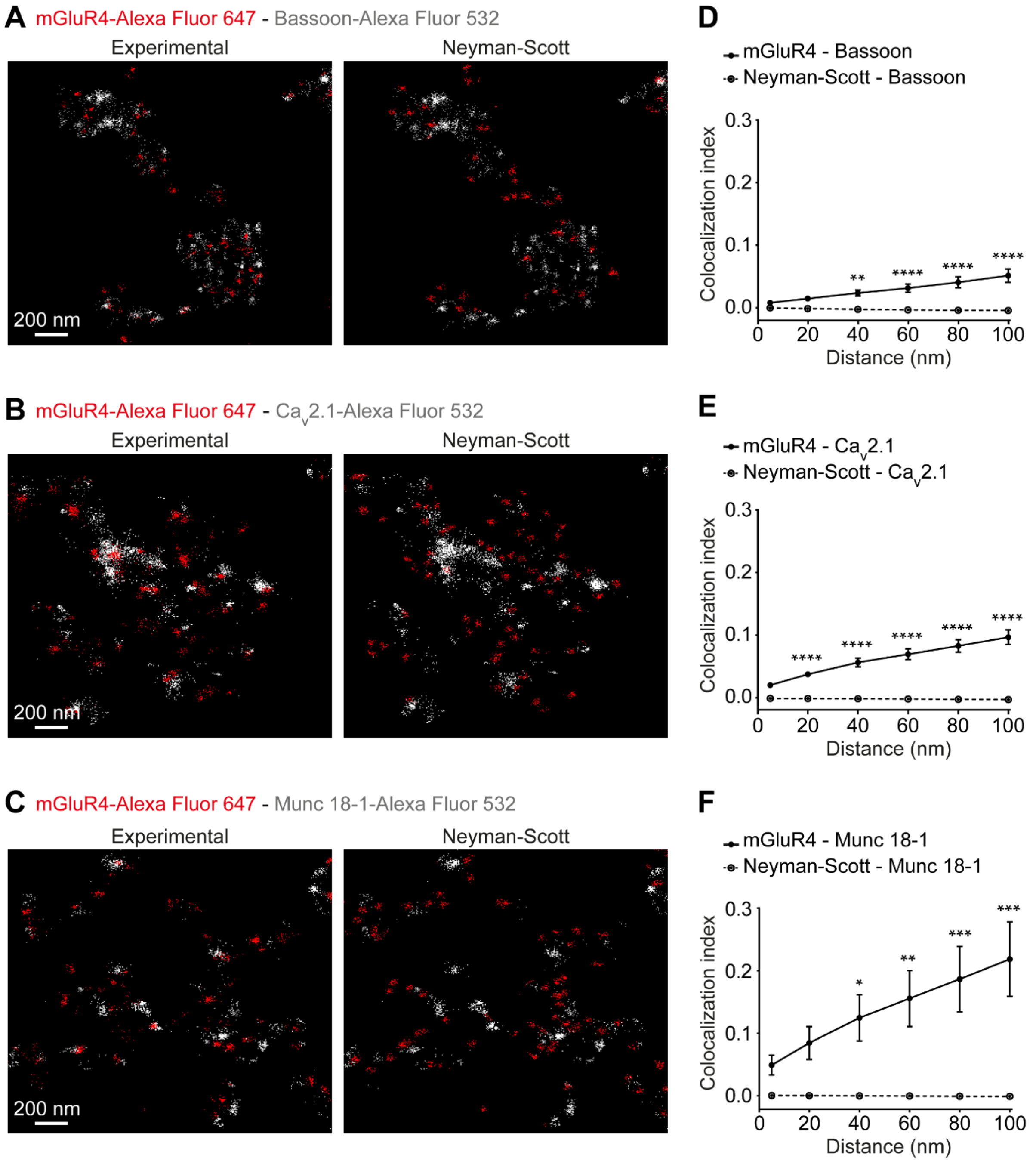

Figure S8. Distance-based colocalization analyses of mGluR4 compared to simulated Neyman-Scott distributions. Distance-based colocalization analyses were performed on data obtained by *d*STORM imaging of cerebellar slices simultaneously stained with two distinct primary antibodies against mGluR4 and bassoon (**A,D**), mGluR4 and Ca$_V$2.1 (**B,E**) or mGluR4 and Munc 18-1 (**C,F**). (**A-C**) Left, two-color images showing mGluR4 localizations (red) and the corresponding localizations in the second channel (gray). Right, same as left panel but with experimental localizations in the first channel replaced by a Neyman-Scott distribution. (**D-F**)

Results of the corresponding distance-based colocalization analyses. Data are are mean ± s.e.m. of 7 (**D**), 10 (**E**) or 3 (**F**) *d*STORM images from two independent preparations co-immunostained for mGluR4 and bassoon or mGluR4 and $Ca_V2.1$ and one preparation co-immunostained for mGluR4 and Munc-18-1, respectively. Differences are statistically significant by two-way ANOVA followed by Holm-Sidak's test. *$P < 0.05$, **$P < 0.01$, ***$P < 0.001$ and ****$P < 0.0001$ versus Neyman-Scott.

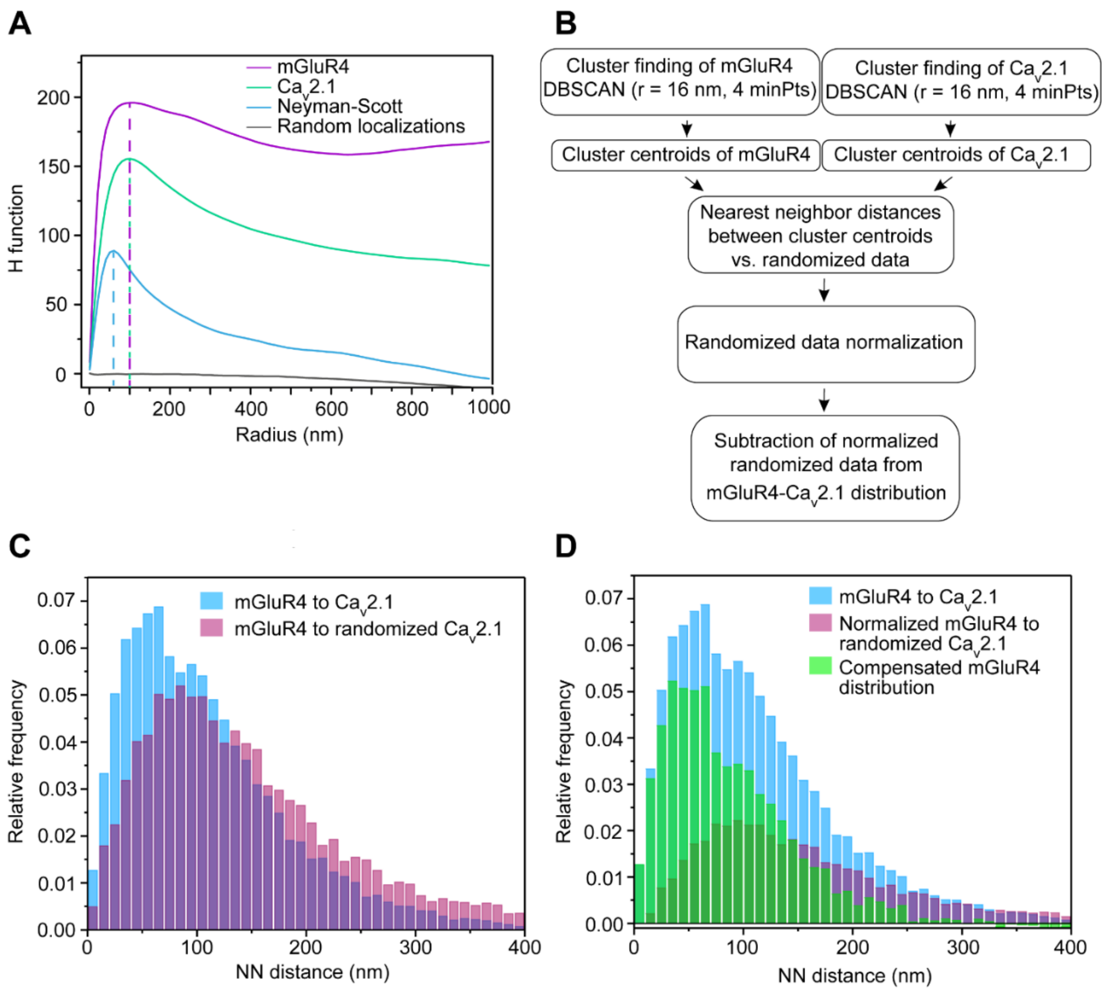

Figure S9. mGluR4 and Ca$_V$2.1 nearest neighbor analysis. (**A**) Ripley's H function analysis investigating the clustering of mGluR4 and Ca$_V$2.1 localizations. Data were compared with a Neyman-Scott distribution (n = 20, σ = 20 nm), used to simulate randomly distributed localization clusters as well as with random uniformly distributed localizations. H maxima were observed at approximately 100 nm (mGluR4), 100 nm (Ca$_V$2.1), and 60 nm (Neyman-Scott). The overlapping of H maxima for mGluR4 and Ca$_V$2.1 justified the use of the same DBSCAN parameters (r = 16 nm, minPts = 4) for both datasets. (**B**) Flowchart of the nearest neighbor (NN) analysis workflow (see Methods for details). (**C**) Results of the NN analysis. The results obtained with randomized Ca$_V$2.1 localizations are given for comparison. (**D**) compensated NN results obtained by subtracting from the NN results the normalized distribution obtained with randomized Ca$_V$2.1 localizations (see methods for details). n = 13,964 distances.

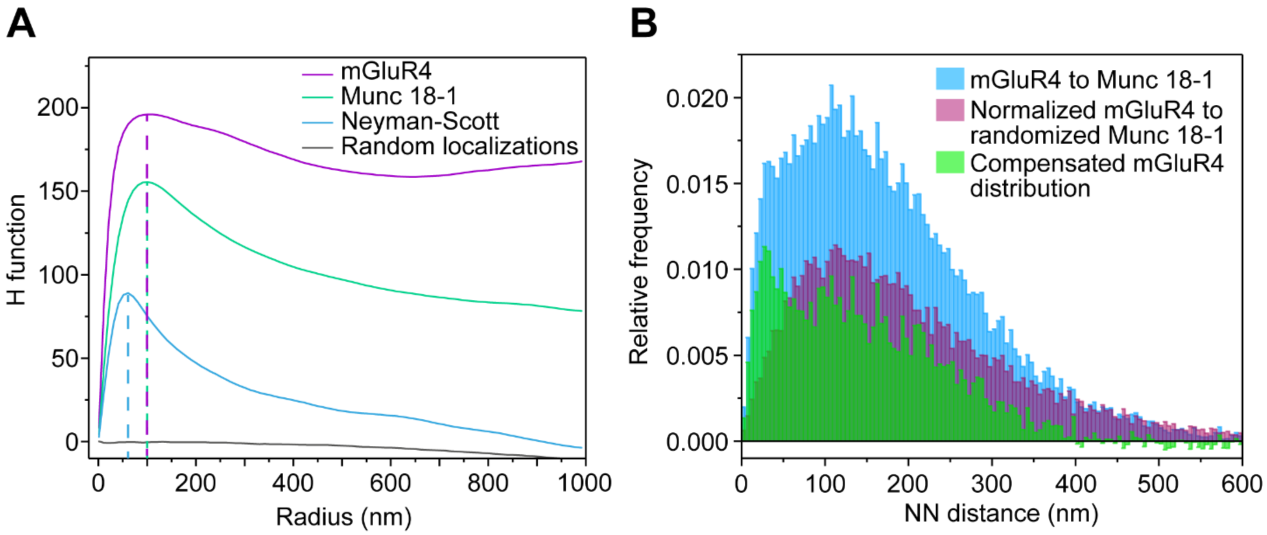

Figure S10. mGluR4 and Munc-18-1 nearest neighbor analysis. (**A**) Ripley's H function analysis investigating the clustering of mGluR4 and Munc-18-1 distributions. Data were compared with a Neyman-Scott distribution (n = 20, σ = 20 nm) to simulate randomly distributed localization clusters as well as with random uniformly distributed localizations. H maxima were observed at approximately 100 nm (mGluR4), 100 nm (Munc-18-1), and 60 nm (Neyman-Scott). (**B**) Results of the NN analysis. Data are shown as in Fig. S8. n = 20,705 distances.
